# Immunomodulatory Role of Mesenchymal Stem Cell Therapy in Multiple Sclerosis: A Systematic Review

**DOI:** 10.7759/cureus.86988

**Published:** 2025-06-29

**Authors:** Maysaa N Amin, Rahma Hashish, Khaled Agha Tabari, Shivling S Swami, Alousious Kasagga, Amanuel Kefyalew Assefa, Ann Kashmer Yu

**Affiliations:** 1 Microbiology and Immunology, California Institute of Behavioral Neurosciences & Psychology, Fairfield, USA; 2 Internal Medicine, Sherwood Forest Hospitals NHS Foundation Trust, Nottingham, GBR; 3 Radiology, Queen Elizabeth University Hospital, Glasgow, GBR; 4 Internal Medicine, California Institute of Behavioral Neurosciences & Psychology, Fairfield, USA; 5 Pathology, Peking University, Beijing, CHN; 6 Orthopaedics and Trauma, University Hospitals of Leicester NHS Trust, Leicester, GBR

**Keywords:** biomarkers, cerebrospinal fluid, immunomodulation, mesenchymal stem cell, multiple sclerosis

## Abstract

The progressive immune-mediated disease known as multiple sclerosis (MS) is characterized by myelin degradation, inflammation, and neurodegeneration. Recent research has examined the potential of mesenchymal stem cell (MSC) therapy for treatment, focusing on its neuroprotective and immunomodulatory properties. This study looked at how MSC transplantation affects cerebrospinal fluid (CSF) biomarkers and the immune system, and checked whether they can indicate the safety and effectiveness of the treatment for MS. According to the Preferred Reporting Items for Systematic Reviews and Meta-Analyses (PRISMA) guidelines, this systematic review was conducted. The final selection comprised eight studies: two randomized controlled trials (RCTs), two non-randomized experimental studies, three systematic reviews with meta-analyses, and one narrative review.

Following MSC therapy in MS patients, significant alterations in neuroprotective biomarker levels were found in the CSF, exhibiting positive results. Immunologically, MSC therapy facilitated the expansion of regulatory T cells (Tregs) and suppressed T helper type 17 (Th17) cell activity, restoring immune balance and diminishing neuroinflammation. Clinically, improvements in Expanded Disability Status Scale (EDSS) scores and MRI lesion burden were observed in a significant subset of patients. Across the included studies, MSC therapy was generally safe, with mild, self-limiting adverse effects. However, heterogeneity in MSC sources, administration routes, and outcome measures, along with small sample sizes, limited comparability. In conclusion, MSC-based therapies show promising potential as a personalized approach to MS management.

## Introduction and background

Multiple sclerosis (MS) represents an inflammatory, immune-mediated disorder of the central nervous system (CNS), characterized by multifocal demyelination and subsequent neuronal degeneration [1]. MS represents the predominant non-traumatic etiology of disability among young adults, affecting more than 2.8 million individuals worldwide and exhibiting a notable rise in incidence in recent decades [1]. Earlier research has demonstrated that immune dysregulation in MS primarily stems from myelin-specific autoreactive CD4+ T cells and is strongly linked to immune dysfunction, temporary activation of immune cells, and an imbalance in immune cell subpopulation ratios [2,3].

Upon activation, naive CD4+ T cells undergo differentiation into T helper type 1 (Th1) and T helper type 2 (Th2) helper subsets, as well as T helper type 17 (Th17) cells and regulatory T (Treg) cells. Significantly, the heightened pro-inflammatory effects of Th17 cells, along with the reduced immunosuppressive ability of Tregs, are essential elements contributing to the breakdown of immune tolerance in MS [4].

Despite notable progress in recent decades, a proven cure remains elusive, and the available treatments have drawbacks that prevent their use in clinical settings due to undesirable side effects [5,6]. At present, there are numerous immunotherapies available, aimed at restoring the balance of Th17/Treg in MS. These include a range of disease-modifying therapies (DMTs), immunosuppressive agents such as interferon beta (IFN-β) [7], glatiramer acetate (GA) [8], teriflunomide, and fingolimod, in addition to several monoclonal antibodies that employ cell depletion strategies [9].

In MS patients, the Th17/Treg imbalance has been somewhat alleviated by these DMTs and other monoclonal antibodies based on cell depletion therapy [10]. However, with a higher risk of infection, tumors, and other negative effects, these medication therapies are nonspecific and suppress the systemic immune system [10,11].

The primary objectives of most currently available therapeutic approaches, which are based on the prescription of immunosuppressive and immune-modulating treatments, are to reduce symptoms, reduce the rate at which disability develops, avoid relapses, and limit disability accumulation [5,6]. Recent studies have shown that mesenchymal stem cells (MSCs) possess strong immunomodulatory potential [12], which makes them extremely promising options for the management of inflammatory autoimmune diseases [13].

MSCs possess significant immunomodulatory properties and exhibit low immunogenicity; they are regarded as a crucial resource for managing the progression of MS and re-establishing immune tolerance. In addition, a deeper understanding of the possible mechanisms behind MSC-mediated Th17/Treg homeostasis is necessary to support the creation of novel MSC-based treatments, targeted at more accurate immune-molecular interventions, and to increase the viability of using MSCs as a type of cell therapy in the clinical treatment of MS [14].

MSCs substantially enhance both innate and adaptive immune responses due to their anti-inflammatory, anti-apoptotic, and immunomodulatory qualities. The immunomodulatory effects may result from direct interactions with immune cells or through the paracrine signaling mechanisms of MSCs [15,16]. These cells belong to a class of non-hematopoietic stem cells (HSCs), distinguished by their ability to self-renew. Since their initial extraction from bone marrow, these cells have been found in several other tissues, including the periendothelium, adipose tissue, epidermis, and umbilical cord blood [17]. They possess a remarkable ability to differentiate into cells representative of diverse mesenchymal lineages, including osteoblasts, chondrocytes, fibroblasts, and adipocytes, when exposed to suitable stimuli [17].

MSCs can display diverse surface markers and cytokine profiles, influenced by their tissue of origin [18]. They play a crucial role in preserving immunological and vascular balance, as well as assisting the bone marrow's HSCs. This is achieved through their capacity to migrate selectively to areas of tissue injury or inflammation - a process known as "homing." MSCs have been the subject of extensive research across various disease models due to their accessibility as a source of either autologous or allogeneic somatic stem cells, which possess the potential to differentiate into multiple cell types [19]. Consequently, novel treatments such as MSC therapy have demonstrated encouraging outcomes in addressing severe MS. By repairing injured nerve cells and halting the progression of the disease, these advanced therapies provide renewed hope for patients who have not adequately responded to traditional treatment methods [6]. Recent RCTs have demonstrated the neuroprotective and immunomodulatory effects of MSC therapy - particularly its impact on cerebrospinal fluid (CSF) biomarkers - which, in turn, influence the progression of the disease [20,21].

Although MSCs are known to modulate immune responses, their exact mechanisms, particularly in regulating the Th17/Treg axis in MS, remain unclear. Addressing these gaps is essential for advancing targeted therapies and improving clinical outcomes. Moreover, several unanswered questions regarding their mechanistic role highlight the need for further research. Therefore, this systematic review aims to synthesize clinical evidence on the immunomodulatory and restorative effects of MSC therapy in MS, with a focus on inflammatory markers, immune balance, and clinical endpoints. This review seeks to clarify the therapeutic potential of MSCs and identify key areas for future investigation.

## Review

Methods

To create this systematic review, we precisely followed the 2020 guidelines for the Preferred Reporting Items for Systematic Reviews and Meta-Analyses (PRISMA) [22].

Eligibility Criteria

We chose the studies according to the elements of Participants, Intervention, and Outcomes (PIO): Participants - MS patients; Intervention - stem cells, MSCs; and Outcome - immune response, an immunomodulatory outcome measure. Furthermore, we implemented additional inclusion and exclusion criteria. The inclusion criteria encompassed English-language full-text articles disseminated within the preceding five years, as well as RCTs, systematic reviews, observational studies, meta-analyses, and reviews. The exclusion criteria comprised case reports, case studies, and editorials.

Selection Process

We imported all relevant articles into EndNote (Clarivate, Philadelphia, PA, USA) and grouped and alphabetized all references using Microsoft Excel 2021 (Microsoft® Corp., Redmond, WA, USA) for duplicate removal. Each identified article underwent a preliminary screening process that involved examining titles and abstracts. Following the identification of eligible articles, we evaluated the availability of their full texts. The most pertinent papers were assessed based on predetermined inclusion and exclusion criteria. We included articles that met all specified criteria in the study. Conversely, we excluded studies that did not provide the necessary analyses for this review. Any disagreements between the reviewers during the study selection or quality assessment phases were initially resolved through discussion; if consensus could not be reached, a third independent reviewer was consulted to reach a final decision. The researchers opted to include systematic reviews, meta-analyses, and narrative reviews in the study, due to the limited number of such works in the field.

Databases and Search Strategy

We systematically searched PubMed, PubMed Central (PMC), Cochrane Library, Google Scholar, and ClinicalTrials.gov for relevant studies published up to March 24, 2025. The search strategy included combinations of Medical Subject Headings (MeSH) and free-text keywords related to “Multiple Sclerosis,” “Mesenchymal Stem Cells,” “Immunomodulation,” and related terms. Boolean operators and database-specific filters (e.g., full text, English language, and last five years) were applied. The complete search strategy is detailed in Table 1.

**Table 1:** Bibliographic search strategy in databases with their corresponding filters.

Databases	Keywords	Search Strategy	Filters	Search Results
PubMed	Multiple Sclerosis disease, Autoimmune diseases, Neurodegenerative diseases, Mesenchymal stem cells (MSCs), Stem cell therapy, Mesenchymal stem cell therapy, Cell and tissue-based therapy, Immune response, Immunomodulatory effect, Autoimmunity	#1 Multiple Sclerosis disease OR Autoimmune diseases OR neurodegenerative diseases OR ("Multiple Sclerosis/blood"[Majr] OR "Multiple Sclerosis/cerebrospinal fluid"[Majr] OR "Multiple Sclerosis/diagnosis"[Majr] OR "Multiple Sclerosis/diagnostic imaging"[Majr] OR "Multiple Sclerosis/diet therapy"[Majr] OR "Multiple Sclerosis/drug therapy"[Majr] OR "Multiple Scler osis/enzymology"[Majr] OR "Multiple Sclerosis/epidemiology"[Majr] OR "Multiple Sclerosis/etiology"[Majr] OR "Multiple Sclerosis/genetics"[Majr] OR "Multiple Sclerosis/history"[Majr] OR "Multiple Sclerosis/immunology"[Majr] OR "Multiple Sclerosis/microbiology"[Majr] OR "Multiple Sclerosis/mortality"[Majr] OR "Multiple Sclerosis/pathology"[Majr] OR "Multiple Sclerosis/prevention and control"[Majr] OR "Multiple Sclerosis/therapy"[Majr] OR "Multiple Sclerosis/urine"[Majr] OR "Multiple Sclerosis/veterinary"[Majr]) #2 Mesenchymal stem cell (MSCs) Stem cell therapy OR mesenchymal stem cell therapy OR ("Mesenchymal Stem Cells/chemistry"[Majr] OR cell and tissue-based therapy OR "Mesenchymal Stem Cells/cytology"[Majr] OR "Mesenchymal Stem Cells/drug effects"[Majr] OR "Mesenchymal Stem Cells/enzymology"[Majr] OR "Mesenchymal Stem Cells/immunology"[Majr] OR "Mesenchymal Stem Cells/metabolism"[Majr] OR "Mesenchymal Stem Cells/microbiology"[Majr] OR "Mesenchymal Stem Cells/pathology"[Majr]) #3 Immune response OR immunomodulatory effect OR autoimmunity OR ("Autoimmunity/drug effects"[Majr] OR "Autoimmunity/genetics"[Majr] OR "Autoimmunity/immunology"[Majr] OR "Autoimmunity/physiology"[Majr] OR "Autoimmunity/radiation effects"[Majr]) #5 #1 AND #2 AND #3	Last Five Years, Free Full Text	231
Cochrane Library	Multiple Sclerosis, Autoimmune disease, Autoimmune disorder, Mesenchymal stem cells (MSCs), Stem cell therapy	#1MeSH descriptor: [Multiple Sclerosis] explode all trees #2 (Multiple sclerosis NEXT (disease)) OR (Autoimmune NEXT (disease) #3 #1 OR #2 #4 MeSH descriptor: [Mesenchymal Stem Cells] explode all trees #5 (Mesenchymal stem cell NEXT (Stem cell therapy)) OR ("stem-cells") #6 #4 OR #5#7 #3 AND #6	2020-2025	19
Google Scholar	Multiple sclerosis disease, Modulation, Mesenchymal stem cell therapy	"Multiple Sclerosis disease" AND "modulation" AND "Mesenchymal stem cell therapy"	2020-2025	122
PMC	Multiple Sclerosis, Mesenchymal stem cells (MSCs), Immune response	#1 Multiple Sclerosis[Title] #2 Mesenchymal stem cells (MSCs) #3 Immune response #4 #1 AND #2 AND #3 (("multiple sclerosis"[MeSH Major Topic] AND ("mesenchymal stem cells"[MeSH Terms] OR ("mesenchymal"[All Fields] AND "stem"[All Fields] AND "cells"[All Fields]) OR "mesenchymal stem cells"[All Fields] OR ("mesenchymal"[All Fields] AND "stem"[All Fields] AND "cell"[All Fields]) OR "mesenchymal stem cell"[All Fields]) AND MSCs[All Fields]) AND ("immunity"[MeSH Terms] OR "immunity"[All Fields] OR ("immune"[All Fields] AND "response"[All Fields]) OR "immune response"[All Fields])	Open Access, Five Years	55
ClinicalTrials.gov	Multiple Sclerosis (MS), Multiple Sclerosis disease, Mesenchymal stem cells (MSCs), Stem cell therapy	"Multiple Sclerosis, MS" AND "Multiple Sclerosis disease” AND “Mesenchymal stem cell (MSCs) STEM CELL THERAPY"	2020-2025	5

Risk of Bias in Individual Studies

We evaluated the remaining full articles for quality and bias risk using specific tools tailored to the type of study by extracting relevant data using a predefined template and implementing commonly employed evaluation tools for each study. We utilized the Cochrane Collaboration Risk of Bias Tool (CCRBT) for RCTs; we employed the Joanna Briggs Institute (JBI) Critical Appraisal Checklist for quasi-experimental studies; we used the Assessment of Multiple Systematic Reviews 2 (AMSTAR 2) for systematic reviews and meta-analyses; and we used the Scale for the Assessment of Narrative Review Articles 2 (SANRA 2) for narrative reviews [23-26]. Each of these assessment tools had distinct criteria and scoring systems. A score of one point was awarded for ratings of "LOW RISK," "YES," "PARTIAL YES," or "1," while two points were assigned for a score of "2." A minimum score of 70% was required for acceptance from each assessment tool (Table 2). A >70% threshold was selected to ensure inclusion of studies with acceptable methodological quality, consistent with practices in prior systematic reviews.

**Table 2:** Assessment of the quality associated with each category of investigation. CCRBT, Cochrane Collaboration Risk of Bias Tool; RCTs, Randomized Controlled Trials; RoB, Risk of Bias; JBI, Joanna Briggs Institute; AMSTAR 2, Assessment of Multiple Systematic Reviews 2; SANRA 2, Scale for the Assessment of Narrative Review Articles 2; PICO, Patient, Intervention, Comparison, and Outcome

Quality Assessment Tool	Type of Study	Items & Their Characteristics	Total Score	Accepted Score (>70%)	Accepted Studies
CCRBT [23]	RCTs	The seven identified factors include the generation of random sequences and allocation concealment (which pertains to selection bias), selective reporting of outcomes (related to reporting bias), various other sources of bias, the blinding of both participants and personnel (associated with performance bias), the blinding of outcome assessment (linked to detection bias), and issues with incomplete outcome data (referred to as attrition bias). Each type of bias is evaluated as having a LOW RISK, HIGH RISK, or UNCLEAR classification.	7	5	Petrou et al. (2022) [20]; Harris et al. (2024) [21]
JBI [24]	Quasi experimental	Nine key considerations: (1) Does the study clearly define the "cause" and the "effect"? (2) Were participants involved in any comparable groups? (3) Did the participants experience comparable treatment or care besides the intervention or exposure under investigation? (4) Was a control group present? (5) Were there multiple assessments of the outcome, both before and after the intervention or exposure? (6) Was the follow-up thorough, and if not, were the discrepancies in follow-up between the groups sufficiently detailed and examined? (7) Were the outcomes of participants in any comparisons assessed consistently? (8) Were the outcomes measured reliably? (9) Was the statistical analysis conducted appropriately? Responses were categorized as YES, NO, UNCLEAR, or NOT ACCEPTABLE.	9	7	Cohen et al. (2023) [27]; Jamali et al. (2024) [28]
AMSTAR 2 [25]	Systematic review, Meta-analysis	Sixteen segments were comprised in total: (1) Did the inclusion criteria, along with the study questions for the review, cover the PICO components? (2) Did the review report explicitly say that the procedures used for the review were decided upon before the review's conduct, and did it explain any notable departures from the protocol? (3) Did the review's authors explain the study designs they decided to analyze? (4) Did the review writers employ a thorough approach to finding relevant literature? (5) Were studies chosen twice by the panel of authors? (6) Were data extracted by the review's authors more than once? (7) Did the reviewers explain why the studies were eliminated? (8) Were the included studies adequately described by the review researchers? (9) To determine the risk of bias (RoB) in each of the individual studies that were part of this assessment, did the review authors follow an appropriate methodology? (10) Were the funding sources for the research that were part of the review disclosed by the review authors? (11) If a meta-analysis was needed, did the review authors use appropriate methods for statistically integrating the results? (12) Did the review's authors discuss how RoB in individual studies probably affected the meta-analysis's or additional synthesis's findings, and if any should be considered? (13) When examining or discussing the review's findings, did the authors consider individual studies' RoB? (14) Were any heterogeneities observed in the review's observations sufficiently analyzed and explained by the review authors? (15) Did the review's authors adequately explore publication bias (slight study bias) and disclose its possible impact on the review's findings if they conducted a quantitative synthesis? (16) Were any possible conflicts of interest disclosed by the review authors, such as any financing they obtained to conduct the review? Rated as either YES or NO. A partial "yes" was taken into account.	16	12	Nawar et al. (2024) [29]; Islam et al. (2023) [6]; Zeng et al. (2022) [30]
SANRA 2 [26]	Narrative review	Six items, including a description of the literature search, referencing, scientific logic, a statement of specific objectives or questions, a justification of the article's relevance to the readership, and an ideal data presentation, scored 0-1 or 2.	12	9	Hu et al. (2024) [14]

Data Collection, Items, and Analysis

This systematic review describes these trials and reviews based on their outcomes, applicability, and limitations, using a narrative synthesis rather than performing a meta-analysis because of the inter-variability among studies, including the heterogeneity of participants, the variety of interventions, and the range of outcome measures. Full articles were read, examined, and tallied for reviews and clinical trials. First author, year, study type, disease, inclusion and exclusion criteria, results, key findings, and funding sources were among the items collected from each study. We added the sample size and demographics of research participants for clinical trials. The reviews addressed the total number of participants and the variety, quantity, and type of studies.

Results

The initial number of relevant studies identified on PubMed, PubMed Central, Cochrane Library, Google Scholar, and ClinicalTrials.gov was 432. EndNote, in total, removed 14 duplicated articles, removed six records by automated tools, and removed four for other reasons. We performed an extensive review of 408 articles, which included a thorough examination of titles and abstracts, as well as an evaluation of full-text accessibility. As a result, 39 articles were chosen for retrieval, while 369 were eliminated from further consideration. After further assessment of the chosen studies, it was determined that 11 reports were not retrievable. Ultimately, we assessed 28 pertinent articles for eligibility and quality based on established appraisal criteria.

**Figure 1:**
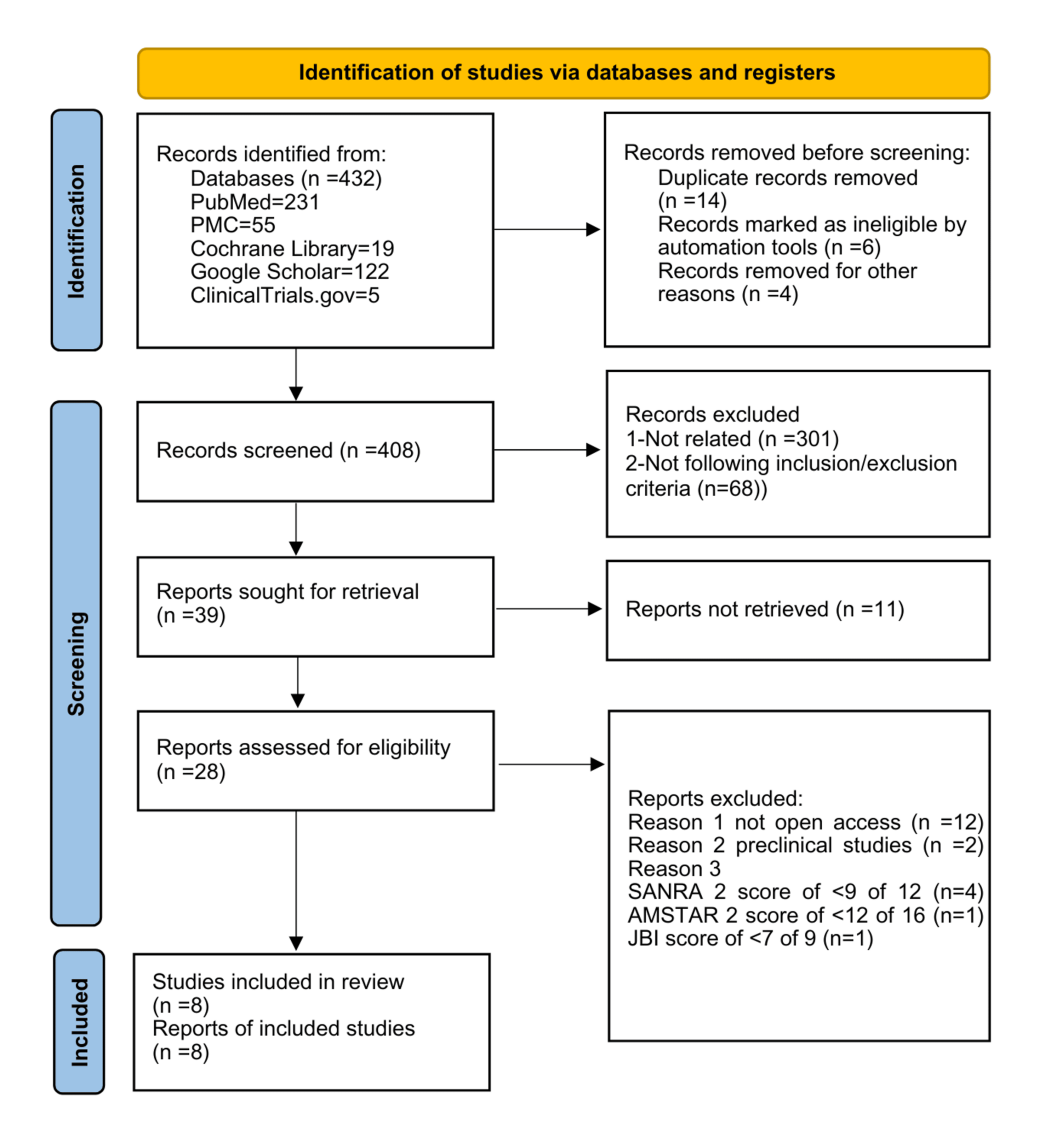
Flowchart showing the article selection process. AMSTAR 2, Assessment of Multiple Systematic Reviews 2; SANRA 2, Scale for the Assessment of Narrative Review Articles 2; JBI, Joanna Briggs Institute

Accordingly, we excluded six studies with scores lower than 70%, 12 studies not open access, and two preclinical studies. As a result, we incorporated eight pertinent articles into the systematic review. Articles with favorable and unfavorable outcomes were selected to mitigate the risk of bias. Figure 1 illustrates the PRISMA flowchart depicting the selection and screening methodology.

The following tables show how each study was evaluated using the corresponding quality assessment tool for each type of study. All RCTs assessed in the review, used the CCRBT and exhibited a "LOW RISK" of bias concerning generating random sequences.

The JBI Critical Appraisal Checklist for quasi-experimental studies (non-randomized experimental studies) was used to assess two quasi-experimental studies in the review [24]. Item 4 was recorded as "NO" for those two studies because there was no control or comparator (Table 3).

**Table 3:** Quality appraisal, Joanna Briggs Institute (JBI) Checklist for quasi-experimental studies (non-randomized experimental studies). "Yes" indicates that the study satisfies the assessment criteria; "No" indicates that it does not; "?" indicates that the information is unclear; and "N/A" indicates that the information is not applicable, according to the Joanna Briggs Institute (JBI).

Criteria	Cohen et al. (2023) [27]	Jamali et al. (2024) [28]
1. Does the study identify the "cause" and the "effect" (i.e., which variable comes first)?	Yes	Yes
2. Did any similar comparisons involve the participants?	N/A	Yes
3. Besides the exposure or intervention of interest, did the participants participate in any comparisons receiving the same care or treatment?	N/A	Yes
4. Was a control group used?	No	No
5. Did the outcome undergo repeated measurements before and after the exposure or intervention?	Yes	Yes
6. Was follow-up thorough, and if not, were follow-up discrepancies between groups appropriately justified and examined?	Yes	Yes
7. Were participant results measured similarly in any comparisons?	Yes	Yes
8. Were the results accurately measured?	Yes	Yes
9. Was the proper statistical analysis applied?	Yes	Yes
Total score	7/9 (100%)	8/9 (100%)
Results	Included	Included

In order to assess the possibility of bias in RCTs, the Cochrane Risk of Bias 2 (RoB 2) Tool Scoring was applied to these studies, as shown in Table 4. Selection, performance, attrition, reporting, and other domains were among the five areas where bias was measured as a judgment (high, low, or ambiguous) for certain elements.

**Table 4:** Risk of bias summary of randomized controlled trials by review authors. LR, Low Risk; UN, Unclear; HR, High Risk

First Author, Year	Random Sequence Generation	Allocation Concealment	Blinding of Participants and Personnel	Selective Reporting	Other Bias
Petrou et al. (2022) [20]	LR	UN	LR	UN	LR
Harris et al. (2024) [21]	LR	LR	LR	LR	LR

Three studies were systematic reviews with meta-analysis. Upon scoring using the AMSTAR 2 tool, all accepted reviews had "NO" in Item 10. One review had "NO" in Item 7. These discussed funding sources and heterogeneity, respectively, as presented in Table 5.

**Table 5:** Result summary of critical appraisal for systematic reviews and meta-analyses by review authors. Y, Yes; PY, Partial Yes; N, No

First Author, Year	Item 1	Item 2	Item 3	Item 4	Item 5	Item 6	Item 7	Item 8	Item 9	Item 10	Item 11	Item 12	Item 13	Item 14	Item 15	Item 16
Zeng et al. (2022) [30]	Y	Y	PY	Y	Y	Y	N	PY	Y	N	Y	PY	Y	Y	PY	PY
Islam et al. (2023) [6]	Y	Y	Y	PY	Y	Y	PY	Y	PY	N	Y	PY	PY	Y	Y	Y
Nawar et al. (2024) [29]	Y	PY	Y	PY	Y	Y	PY	PY	Y	N	Y	Y	Y	Y	Y	Y

Finally, Table 6 illustrates the evaluation of narrative reviews utilizing the SANRA 2 checklist, which comprises six criteria. The accepted review received a score of "1" for its literature search description and the suitable presentation of data.

**Table 6:** Result summary of quality assessment of narrative reviews by review authors.

First Author, Year	Justification of the Article’s Importance for the Readership	Statement of Concrete Aims or Formulation of Questions	Description of the Literature Search	Referencing	Scientific Reasoning	Appropriate Presentation of Data
Hu et al. (2024) [14]	2	2	1	2	2	1

Study Characteristics

The following tables show, chronologically, the main characteristics of clinical trial studies (RCT and non-RCTs) and reviews (systematic reviews, meta-analyses, and narrative reviews). Of the eight studies accepted in the review, three articles focused on a population of progressive MS patients (Secondary progressive multiple sclerosis (SPMS) and primary progressive multiple sclerosis (PPMS)), while five studies examined all MS patients (including relapsing-remitting multiple sclerosis (RRMS), SPMS, and PPMS).

There were 157 participants with MS in the RCTs and non-RCTs. Forty-eight patients received MSC transplantation, either intrathecal (IT) or intravenous (IV); 20 participants (two dropouts) received three IT administrations of autologous mesenchymal stem cell neurotrophic factor (MSC-NTF) cells; 54 participants (seven dropouts) received six IT injections of autologous mesenchymal stem cell-neural progenitors (MSC-NPs). A total of 35 participants received two treatment protocols involving the administration of umbilical cord-derived mesenchymal stem cells (UC-MSCs) via IT and IV routes. The study lacked a control group and was designed as a non-RCT with an open-label format. Table 7 shows these findings.

**Table 7:** Main characteristics of the randomized controlled and non-randomized trials accepted in the review. PMS, Progressive Multiple Sclerosis; MS, Multiple Sclerosis; PPMS, Primary Progressive Multiple Sclerosis; SPMS, Secondary Progressive Multiple Sclerosis; MSC, Mesenchymal Stem Cell; IT, Intrathecal; IV, Intravenous; NF-L, Neurofilament Light Chains; CXCL13, CXC Motif Chemokine Ligand 13; CSF, Cerebrospinal Fluid; EDSS, Expanded Disability Status Scale; MSC-NTF, Mesenchymal Stem Cell Neurotrophic Factor; MRI, Magnetic Resonance Imaging; DMT, Disease-Modifying Therapy; BP, Blood Pressure; BM-MSCs, Bone Marrow Derived Mesenchymal Stem Cells; UC-MSCs, Umbilical Cord Derived Mesenchymal Stem Cells

First Author, Year	Study Type	Disease	Inclusion & Exclusion Criteria	Sample Size (Dropouts)	Gender	Age	Intervention	Control Group	MSC Source	Outcomes Measured & Key Points	Funding Sources
Petrou et al. (2022) [20]	Double-blind, randomized phase II clinical trial	PMS	Inclusion: Patients with PMS who participated in the trial; Exclusion: Not mentioned	48 patients	Not Stated	Not Stated	MSC transplantation, either IT or IV	The placebo group received sham treatment.	BM-MSCs	Concentrations of NF-L and CXCL13 in CSF suggest potential neuroprotective effects of MSC transplantation in MS patients.	No external source supported this study, and it was performed using the Investigator’s research grants.
Cohen et al. (2023) [27]	Non-randomized clinical trial	PMS	Inclusion: Males and females aged 18-65 with a clinical diagnosis of PMS, no relapse for 6 months, and an EDSS score of 3.0-6.5; Exclusion: not mentioned	20 participants were screened; 18 were treated (2 dropouts due to adverse events).	10 females and eight males.	18-65 years; Mean age of 47.4 ± 9.6 years.	Three intrathecal administrations of autologous MSC-NTF cells at Weeks 0, 8, and 16	There is no control group; the study is non-randomized and open-label.	BM-MSCs	Safety, tolerability, changes in disability progression, and biomarkers of neuroprotection. No deaths or significant adverse events related to MS were reported.	Brainstorm Cell Therapeutics provided funding, while the National Multiple Sclerosis Society provided support through a grant (award no.: FF181232990).
Harris et al. (2024) [21]	Phase II, randomized, double-blind, placebo-controlled clinical trial with a compassionate crossover design	PMS, specifically SPMS and PPMS	Inclusion: Clinically definite SPMS or PPMS, EDSS score between 3.0 and 6.5, stable disease (less than 1.0 point change in EDSS, no clinical relapses in the past 12 months, stable MRI disease burden), disease duration of less than 20 years, and no change in DMT less than 12 months before trial; Exclusion: Existing medical comorbidities or cancer history that could complicate safety outcomes	54 participants (7 dropouts: 27 in the MSC-NP group and 27 in the saline group); 7 dropouts (3 in year 1 and 4 in year 2).	Female 27, MSC-NP group 27, Saline group	Mean age (years): 51 (7), MSC-NP group; 49 (9), saline group	Six intrathecal injections of autologous MSC-NPs	The saline (placebo) group received six injections.	BM-MSCs	The main element is EDSS Plus, which includes T25FW, EDSS Expand, and the nine-hole peg exam. Secondary: Individual EDSS Plus components, urodynamic testing, brain atrophy measurement, and the six-minute walk test (6MWT). The comparison of the EDSS Plus improvement between saline (37%) and MSC-NP (33%) groups showed no significant difference.	The study was funded by Damial Foundation, Biogen, and the National Multiple Sclerosis Society.
Jamali et al. (2024) [28]	Non-randomized phase I/II clinical trial	MS	Inclusion criteria: Men and women at least 18 years old are eligible for inclusion. When getting involved, written informed consent was collected. All forms of MS, Mini-Mental State Examination score ≥ 24, EDSS score < 6.5, and independent in walking, even with an assistive device. Criteria for exclusion: untreated visual loss, history of a condition affecting the neurological system other than MS, severe disabilities of the body, brain, or senses that would seriously disrupt testing, developmental background of attention-deficit/hyperactivity disorder or learning disability, corticosteroid usage or relapse within four weeks of evaluation, coronary artery bypass surgery or an acute ischemic cardiovascular episode within three months, medication-uncontrolled BP more than 190/110 mmHg	35 participants	Group A had eight females and 12 males. Group B had 12 females and three males.	Average age: Group A 37.25 and Group B 35.8	Two treatment protocols of UC-MSCs, IT, and IV.	Group A received two intrathecal doses of UC-MSCs, while Group B received a single dose.	UC-MSCs	The outcomes assessed encompassed a general disability scale, lesion load, cortical thickness, manual dexterity, and information processing speed. Both protocols demonstrated safety, with Group A exhibiting significant enhancements compared to Group B (p-value < 0.05).	The Hashemite Kingdom of Jordan's Ministry of Higher Education's Scientific Research Fund funded this study under grant number MPH/1/38/2017.

There were 9, 18 RCTs, 30 studies in systematic review, 22 studies in meta-analysis, and various clinical trials (phase I/II, pilot studies without exact number) in the four reviews, respectively. These reviews provided explicit inclusion and exclusion criteria for MS patients and autoimmune diseases; outcomes were detailed accordingly, and all research involved individuals diagnosed with MS according to established MS diagnostic criteria. Funding sources differed with each study. Table 8 presents these findings.

**Table 8:** Key features of the systematic reviews, meta-analyses, and narrative reviews included in the review. RCTs, Randomized Controlled Trials; RA, Rheumatoid Arthritis; SLE, Systemic Lupus Erythematosus; MSC, Mesenchymal Stem Cell; SCT, Stem Cell Transplantation; EDSS, Expanded Disability Status Scale; MS, Multiple Sclerosis; AHSCT, Autologous Hematopoietic Stem Cell Transplantation; STDF, Science, Technology, and Innovation Funding Authority; EKB, Egyptian Knowledge Bank; RRMS, Relapsing-Remitting Multiple Sclerosis; SPMS, Secondary Progressive Multiple Sclerosis; PPMS, Primary Progressive Multiple Sclerosis; CI, Confidence Interval

First Author, Year	Study Type	No. & Type of Included Studies	Total Participants, Range	Inclusion & Exclusion Criteria	MSC Source	Outcomes	Key Points	Funding Sources
Zeng et al. (2022) [30]	Systematic review and meta‑analysis of randomized controlled trials	18 RCTs focusing on five autoimmune diseases (the five autoimmune diseases were RA, SLE, inflammatory bowel disease, ankylosing spondylitis, and multiple sclerosis).	Not specified	Inclusion criteria: Patients diagnosed with any autoimmune disease according to recognized standards; no restrictions on gender, age, or region. Exclusion criteria: (1) animal experiments, (2) not RCT, (3) basic research, and (4) the intervention of the control group was MSC transplantation.	Does not specify a single dominant MSC source.	Efficacy and safety indicators of MSC transplantation in treating specified autoimmune diseases.	MSC transplantation may reduce disease activity and improve clinical symptoms; no significant adverse events were reported.	Not disclosed
Islam et al. (2023) [6]	Systematic review and meta-analysis	30 studies in a systematic review, 22 studies in a meta-analysis	Not specified	Inclusion: Clinical studies on human subjects aged 18 or above and studies reporting efficacy and safety of MSC therapy in human MS patients based on EDSS score changes. Exclusion: Meeting abstracts, review articles, case reports, non-human studies, theses, and opinions. ​	Heterogeneous MSC origins (bone marrow, umbilical cord, and adipose tissue).	Changes in the EDSS score from baseline to follow-up.	40.4% (95% CI: 30.6-50.2) of MS patients showed improvement, 32.8% (95% CI: 25.5-40.1) remained stable, and 18.1% (95% CI: 12.0-24.2) experienced worsening after MSC therapy. Minor adverse events like headaches 57.6 (37.9-77.3) and fever 53.1 (20.7-85.4) were reported, but no significant complications were observed.	No external funding
Nawar et al. (2024) [29]	Systematic review and meta-analysis of randomized controlled trials	9 RCTs	422 participants, 9 to 144.	Inclusion: RCTs of SCT in MS patients. Exclusion: Observational studies, non-English studies, animal or experimental trials, reviews, book chapters, conference abstracts, trial registries, and protocols.​	Does not specify a single dominant MSC source.	SCT significantly improved EDSS at 2 months (95% CI: − 1.08, -0.06, p = 0.03) and reduced brain lesion volume (95% CI: -10.69, -3.4, p = 0.0002); non-significant effect on EDSS at 6 and 12 months, T25-FW, and brain lesion number; significant adverse events included local reactions at the MSC infusion site (95% CI: 1.08, 6.03, p = 0.034) and hematological disorders after AHSCT (95% CI: 1.23, 4.39, p = 0.009).	SCT has both remyelinating and immunomodulatory characteristics in people with multiple sclerosis. This review assesses SCT's clinical usefulness and safety, pointing out that transplantation protocols - including dosage, cell source, and administration route - vary between trials.	Open access financing supplied by the STDF in collaboration with the EKB.
Hu et al. (2024) [14]	Narrative review	Various clinical trials (phase I/II, pilot studies) on MSC therapy for MS (exact number not specified)	Not specified	Inclusion criteria: Patients with RRMS, SPMS, and PPMS; some trials included those who failed conventional treatments. Exclusion criteria: Not explicitly stated, but likely included patients with severe comorbidities or contraindications to MSC therapy.	Does not specify a single dominant MSC source.	MSCs alter the differentiation of CD4+ T cells, inducing anergy and reducing Th17 cells. Polarization of antigen-specific Tregs to reverse Th17/Treg imbalance. Reduction in inflammatory responses and restoration of immune tolerance.	MSCs regulate Th17/Treg homeostasis through various mechanisms, including extracellular vesicles, metabolic reprogramming, and autophagy. The goal of the review is to identify new targets for MS treatment using cellular therapy, with a focus on the mechanisms and outcomes related to MSCs and their impacts on immune regulation in MS.	National Natural Science Foundation of China (No. U2004128).

Discussion

Neurodegeneration represents a fundamental feature of MS, characterized by axonal damage, myelin loss, and neuronal degeneration. MSC therapy has shown potential not only for modulating immune responses but also for offering neuroprotective benefits. This section examines how MSC transplantation affects CSF biomarkers related to neuroprotection and assesses their utility as indicators of treatment efficacy in MS. These biomarkers encompass both pro-inflammatory and anti-inflammatory markers. The reviewed studies highlight that MSCs promote the development of Tregs and inhibit pro-inflammatory Th17 cells, helping restore immune homeostasis. This systematic review concluded that MSC therapy is generally safe and well tolerated, and leads to improvements in disability levels and a reduction in lesion burden for a significant number of individuals with MS.

Impact of MSC Transplantation on CSF Neuroprotective Biomarkers

Neurofilament light chains (NF-L) and CXC motif chemokine ligand 13 (CXCL13) have been demonstrated to function as dependable biomarkers for both inflammatory processes and neurodegenerative changes in the context of MS. Elevated concentrations of NF-L have been consistently identified in the CSF of individuals diagnosed with MS, indicating that NF-L may serve as a significant biomarker for assessing disease activity, as well as the efficacy of various therapeutic interventions for MS [31]. One study showed that CSF concentrations of NF-L were significantly diminished six months after treatment with MSCs in patients diagnosed with progressive MS (p-value <0.05). CXCL13 concentrations were also lower; however, this reduction did not reach statistical significance. MSC transplantation may have a neuroprotective effect on MS patients, according to the study’s findings [20]. In contrast, another study found no changes in CSF-NfL levels after IT MSC injections, which contrasts with the previously mentioned study that found decreased NfL levels [21]. This contradiction requires larger-scale studies with a more extended follow-up to confirm these observations. Differences in CSF-NfL findings may be attributed to several factors, including variation in MSC source (e.g., autologous bone marrow vs. umbilical cord vs. MSC-NP), dose and frequency of administration, and the route of delivery (IT vs. IV). Additionally, the timing of post-treatment sampling and patient-specific disease characteristics (e.g., disease stage or baseline inflammatory activity) could influence biomarker dynamics and partially explain these inconsistencies. These considerations highlight the need for standardized protocols in future studies.

Furthermore, the study revealed elevated levels of matrix metalloproteinase-9 (MMP9) and reduced levels of chemokine ligand 2 (CCL2) in the CSF following treatment with intrathecal mesenchymal stem cell-neural progenitors (IT MSC-NPs) in cases of progressive multiple sclerosis (PMS), indicating possible neuroprotective properties and the modulation of neuroinflammatory responses. Elevated MMP9 post-MSC-NP treatment may target cells involved in CNS repair and regeneration, while decreased CCL2 levels indicate a reduction in neuroinflammation, possibly influencing disease progression [21]. Remarkably, after fetal neural stem cell transplantation (SCT), there was also an increase in CSF MMP9 levels in MS patients [32]. The regenerative response linked to IT cell therapy is further implied to be biomarked by MMP9 [21]. Furthermore, the increase of MMP9 and the decrease of CCL2 in the CSF following MSC-NP therapy act as biochemical indicators of the treatment, suggesting its potential efficacy in patients with MS.

According to another recent study, treatment with autologous MSC-NTF-secreting cells in PMS led to a noteworthy increase in CSF neuroprotective factors like vascular endothelial growth factor A (VEGF-A), hepatocyte growth factor (HGF), neural cell adhesion molecule 1 (NCAM-1), follistatin, leukemia inhibitory factor (LIF), and fetuin-A, and a reduction in inflammatory biomarkers such as monocyte chemoattractant protein-1 (MCP-1), stromal cell-derived factor 1 (SDF-1), osteopontin, and CD27 [27]. These findings suggest a potential therapeutic benefit that warrants further investigation through randomized studies to explore the utility of CSF neurodegenerative biomarkers as outcome measures in PMS treatment evaluation, as the limited sample size may influence the assessment of these biomarkers [27].

**Figure 2:**
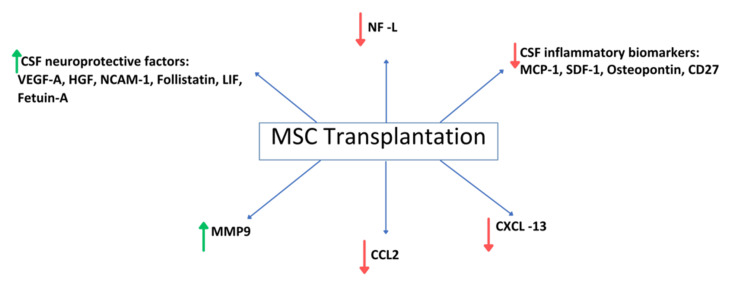
Alterations in CSF biomarkers following MSC transplantation. MSC, Mesenchymal Stem Cell; CSF, Cerebrospinal Fluid; NF-L, Neurofilament Light Chains; VEGF-A, Vascular Endothelial Growth Factor A; HGF, Hepatocyte Growth Factor; NCAM-1, Neural Cell Adhesion Molecule 1; LIF, Leukemia Inhibitory Factor; MCP-1, Monocyte Chemoattractant Protein-1; SDF-1, Stromal Cell-Derived Factor 1; CXCL13, CXC Motif Chemokine Ligand 13; MMP-9, Matrix Metalloproteinase-9; CCL2, Chemokine Ligand 2

Neurodegenerative and inflammatory biomarkers have provided essential biological insights into PMS, with CSF biomarkers indicating inflammation in the CNS [33,34]. In addition to these preliminary results, examining immunosoluble factors in the CSF of individuals with PMS revealed two categories of immune mediators. One group was characterized by pro-inflammatory activities, including IFN-γ (interferon gamma), MCP-1, TNF-α (tumor necrosis factor alpha), MIP-1α (macrophage inflammatory protein-1 alpha), MIP-1β (macrophage inflammatory protein-1 beta), IL-8 (interleukin-8), and IP-10 (interferon gamma-induced protein 10), while the other comprised anti-inflammatory factors such as IL-9, IL-15, IL-1ra, and VEGF [26]. These findings provide significant insights into the immune mechanisms implicated in PMS, underscoring the need for further investigation of these biomarkers in relation to disease progression, diagnostic accuracy, and predictive responses to treatment. Moreover, significant changes in the biomarkers present in CSF were noted following treatment with MSCs, supporting the clinical findings reported in the literature [21]. Figure 2 illustrates these findings.

Immunomodulatory Mechanisms of MSCs Therapy in MS

MS is an immune-mediated inflammatory condition characterized by dysregulated, pro-inflammatory CD4+ T cells in the CNS, leading to non-traumatic disabilities in young adults [35,36]. A key cellular marker for remission in MS is the increase in CD4+ T cells, which includes Tregs, that play a crucial role in reducing inflammation associated with the disease. A recent study demonstrated that administering UC-MSCs intrathecally to MS patients improved disability scales, lymphocyte counts, and various disease metrics, highlighting the potential of MSC therapy for MS treatment. The results indicated a notable rise in CD4+ T cells, particularly the Treg subset, at 12 months following treatment (p-value < 0.05). In contrast, natural killer (NK) cell levels further declined in these patients, indicating an immune-suppressive effect. This suggests that giving MS patients two doses of treatment may improve their immune responses [28]. Furthermore, larger multisite studies with similar or higher doses of MSCs are needed to validate the findings and optimize therapy protocols for MS treatment.

Moreover, an imbalance between Th17 cells and Tregs plays a significant role in the pathogenesis of MS [37-39]. The breakdown of peripheral immune tolerance triggers the activation of autoreactive CD4+ T cells within the lymph nodes, particularly Th1 and Th17 cells, which then evolve into highly aggressive effector cells, including both Th1 and Th17 phenotypes [35]. In addition, prior research has demonstrated that mitochondrial disorders of CD4+ T cells can alter their metabolic patterns in MS patients. This can result in a disruption of CD4+ T cell subset differentiation, which, in turn, can cause a Th17/Treg skew towards Th17 cells and intensify the inflammatory response in vivo [40]. An alternative viewpoint for investigating the mechanism of MSCs in MS therapy is offered by this pathway. It increases the therapeutic potential of stem cells and helps position MSC-based cell therapy as a cutting-edge approach that targets specific organelles [14].

**Figure 3:**
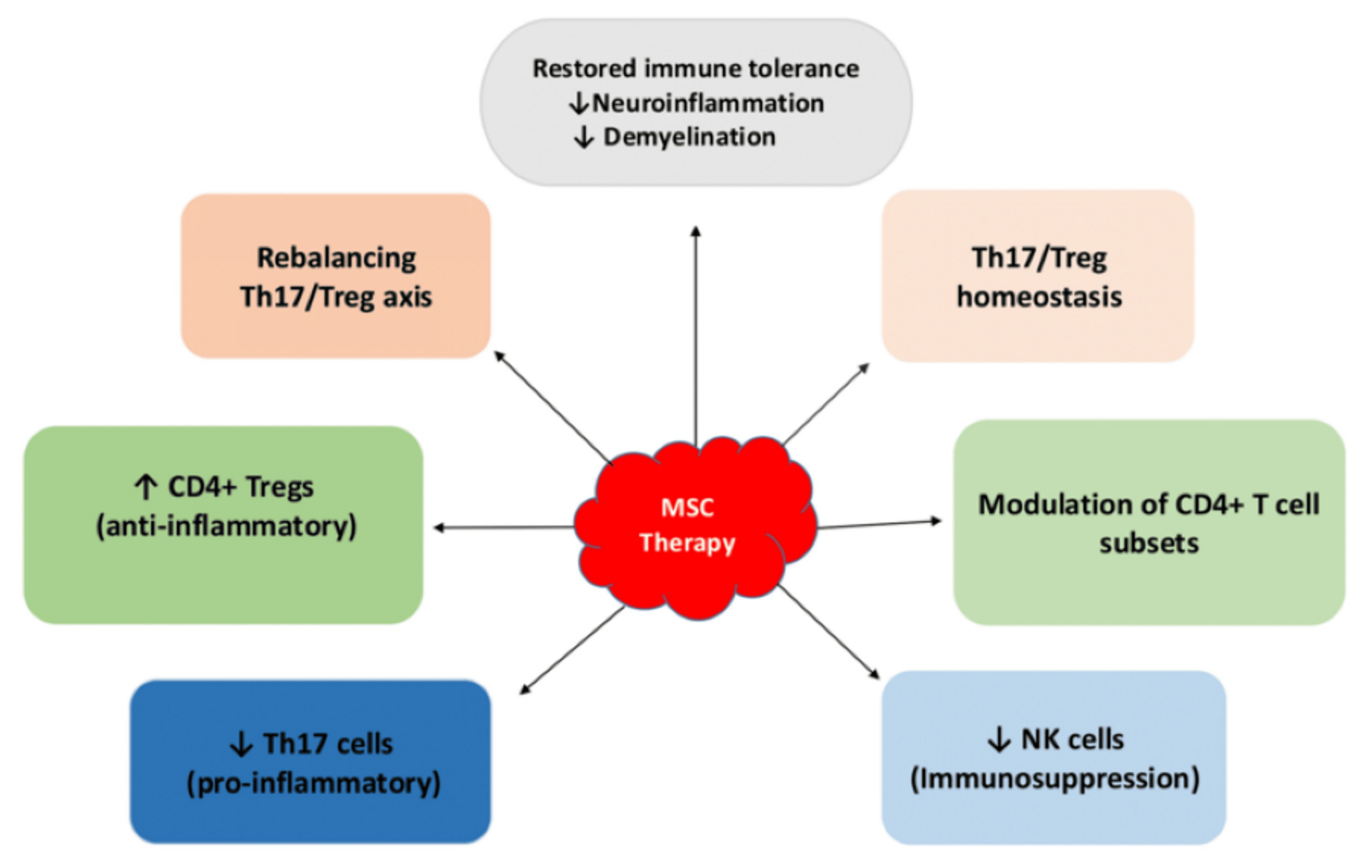
Immunomodulatory mechanism of mesenchymal stem cell therapy. MSC, Mesenchymal Stem Cell; Treg, Regulatory T Cells; NK, Natural Killer Cells; Th17, T Helper 17

Specifically, MSCs inhibit the differentiation of Th17 and Th1 cells, while concurrently promoting the differentiation of Treg cells and influencing macrophage polarization to enhance the formation of anti-inflammatory M2 macrophages in preference to pro-inflammatory M1 macrophages [15,16]. Furthermore, MSC injection affects the differentiation of CD4+ T cells. This effect is evident in the induction of anergy and a reduction in Th17 cell populations, which facilitates the polarization of antigen-specific Treg cells. Such a mechanism aids in rectifying the imbalance within the Th17/Treg axis, consequently reducing the inflammatory response and demyelination, while promoting an overall state of immune tolerance [14]. Apart from the above, the study showed that MSC therapy regulates Th17/Treg homeostasis via extracellular vesicles, metabolic reprogramming, mitochondrial transfer, and autophagy. These pathways are essential for restoring immune self-stabilization and tolerance, thereby reducing neuroinflammation and demyelination linked to MS. Considering the significant relationship between cellular metabolism and immunoregulatory networks, compounds that facilitate mitochondrial translocation and metabolic reprogramming could represent valuable targets for MSCs in the regulation of immune homeostasis, as well as for identifying novel therapeutic avenues for MS via cellular therapy [14]. However, MSC therapy remains in an early investigational stage, with further clinical studies needed to validate its safety and therapeutic efficacy. The flowchart in Figure 3 summarizes the immunomodulatory mechanism of MSC therapy.

Safety, Efficacy, and Clinical Outcomes of MSC Therapy

Clinical trials indicate that SCT can lead to significant clinical recovery and enhanced quality of life for patients with MS, with few safety issues reported [41,42]. A recent study evaluated the effectiveness and safety of SCT for MS, incorporating data from nine RCTs with 422 participants. The results indicated that SCT significantly surpassed the control group in enhancing patients' Expanded Disability Status Scale (EDSS) scores at the two-month interval (p-value = 0.03) and in decreasing the volume of MRI-T2-weighted lesions (p-value = 0.0002) [29]. The procedure was largely safe and well-tolerated, with no reported fatalities or major serious adverse events, aside from infusion site reactions following MSC therapy during the follow-up period. Patients receiving MSC therapy did report localized adverse events at the infusion site. According to these findings, SCT may help people with MS reduce their brain lesion volume and lessen their disability, while maintaining a generally acceptable safety profile. However, further research is required to confirm these findings and evaluate their long-term implications [29].

Similarly, one study evaluated 18 RCTs to investigate whether MSC transplantation is safe and effective in treating a range of autoimmune diseases, including MS [30]. MSCs may have a positive influence on several autoimmune conditions, according to these findings, potentially decreasing disease activity and improving clinical symptoms. Importantly, no significant adverse events associated with MSC transplantation were reported, indicating the generally safe nature of this treatment. However, the results concerning MS were less definitive; while some studies indicated symptom improvements, inconsistencies in dosage and administration techniques led to controversial overall outcomes, underlining the necessity for further research [30]. Additionally, this research offers promising insights into the use of MSCs for treating autoimmune diseases, highlighting potential benefits and safety, while emphasizing the need for further RCTs to refine or confirm these conclusions.

The results of a similar study assessed the changes in EDSS scores from baseline to follow-up and revealed that around 40.4% (95% CI: 30.6-50.2) of MS patients experienced improvement following MSC therapy, while 32.8% (95% CI: 25.5-40.1) remained stable, maintaining their condition, and 18.1% (95% CI: 12.0-24.2) of patients saw a decline in their health. Although no significant complications were reported, some minor adverse effects, such as headaches and fever, were observed. These findings suggest that a significant proportion of MS patients experienced benefits from MSC therapy, underscoring its potential as a viable treatment option [6]. Furthermore, the lack of significant adverse events suggests this therapeutic strategy is safe for MS patients. This study offers important insights into the effectiveness and safety of MSC therapy in managing MS, presenting a comprehensive understanding of its benefits for individuals with this condition [6]. Nevertheless, more studies, technological advancements, MSC dose optimization, and larger clinical trials are crucial for a comprehensive assessment of the long-term effectiveness and safety profiles of MSC therapy in MS patients.

Limitations

This systematic review thoroughly followed PRISMA 2020 guidelines. The study encompasses a combination of recent RCTs, non-RCTs, narrative reviews, systematic reviews, and high-quality meta-analyses. We comprehensively searched the literature through databases, including studies published in the last five years (from 2020 to 2025), to find more recent research and create precise results. We also included grey literature and other databases. We could disregard past research that might have relevant findings.

Despite the encouraging findings, which revealed statistically significant results emphasizing the safety benefits of MSCs as a neuroprotective and immunomodulatory potential therapy in MS patients, several limitations constrain the interpretation and generalizability of the current evidence. First, sample size limitations were a significant concern in many of the included studies. Second, the heterogeneity of MSC preparations and administration protocols complicates direct comparisons of studies. Various studies have used different MSC sources (bone marrow, umbilical cord, or autologous MSC-NTF), delivery routes (IT vs. IV), and dosing schedules, which may account for the conflicting outcomes. Third, the short follow-up durations in many trials limit the understanding of the long-term effects and sustainability of clinical and biomarker improvements. Additionally, the lack of double-blinded, placebo-controlled designs in several trials introduces a risk of bias, particularly in subjective outcome measures, such as the EDSS.

In addition, the absence of a formal assessment of publication bias was primarily due to the heterogeneous nature of the included studies, which varied in design, size, and outcome reporting. Nevertheless, this represents a potential source of bias, as studies with negative results may be underrepresented in the published literature. Future systematic reviews with larger sample sizes and more homogeneous datasets should incorporate publication bias analyses to better evaluate the robustness of reported effects. Moreover, variations in follow-up duration and study heterogeneity may result in inconsistent conclusions. The review's limitations underscore the need for future research to undertake larger, standardized, multi-center trials with extended follow-up and thorough safety evaluations.

## Conclusions

In conclusion, the studies included in this review show that exploring MSC therapy for MS has revealed a significant array of encouraging findings related to its neuroprotective and immunomodulatory effects. A comprehensive systematic review of the existing literature highlights that the transplantation of MSCs has a major impact on CSF biomarkers related to neuroprotection, including NF-L and CXCL13. These biomarkers are vital markers of inflammation and neuronal health, indicating that MSC therapy may be important for preserving the integrity and functionality of the brain. Additionally, MSCs promote immune balance by modulating T cell populations, which is vital in a condition characterized by immune-mediated damage. Initial findings regarding the safety and effectiveness of MSC therapy are promising, indicating improvements in disability and reductions in lesion burden. This growing body of research holds substantial promise for developing innovative treatment strategies tailored for individuals affected by MS. By addressing the neurodegenerative features and the immune dysregulation associated with the disease, MSC therapy could enhance intervention effectiveness and improve the quality of life for affected individuals. Future suggestions for this study include conducting additional research, prioritizing the standardization of MSC treatment protocols, and implementing larger, RCTs with longer follow-up durations. Such studies are crucial for validating the therapeutic potential of MSCs in MS, thereby providing a clearer understanding of their long-term effects and mechanisms of action.
